# The VraSR regulatory system contributes to virulence in *Streptococcus suis* via resistance to innate immune defenses

**DOI:** 10.1080/21505594.2018.1428519

**Published:** 2018-04-24

**Authors:** Peixi Chang, Weitian Li, Guolin Shi, Huan Li, Xiaoqing Yang, Zechen Xia, Yuan Ren, Zhiwei Li, Huanchun Chen, Weicheng Bei

**Affiliations:** a State Key Laboratory of Agricultural Microbiology, College of Veterinary Medicine, Huazhong Agricultural University, Wuhan, China; b Key Laboratory of Preventive Veterinary Medicine in Hubei Province, The Cooperative Innovation Center of Sustainable Pig Production, Huazhong Agricultural University, Wuhan, China; c Huazhong Agricultural University hospital, Huazhong Agricultural University, Wuhan, China; d College of Food Science and Technology, Huazhong Agricultural University, Wuhan, China

**Keywords:** VraSR, polymorphonuclear leukocytes, *Streptococcus suis*, two-component regulatory system, virulence

## Abstract

*Streptococcus suis* is a highly invasive pathogen that can cause sepsis and meningitis in pigs and humans. However, we have limited understanding of the mechanisms *S. suis* uses to evade innate immunity. To investigate the involvement of the two-component signal transduction system of *S. suis* in host immune defense, we examined the expression of 15 response regulators of *S. suis* following stimulation with polymorphonuclear leukocytes (PMNs). We found that several response regulators were significantly up-regulated including *vraR*. Thus, we constructed an isogenic deletion mutant of *vraSR* genes in *S. suis* and demonstrated VraSR promotes both bacterial survival in human blood and resistance to human PMN-mediated killing. The VraSR mutant was more susceptible to phagocytosis by human PMNs and had greater sensitivity to oxidant and lysozyme than wild-type *S. suis*. Furthermore, *in vitro* findings and *in vivo* evidence from a mouse infection model together strongly demonstrate that Δ*vraSR* had greatly attenuated virulence compared with wild-type *S. suis*. Collectively, our data reveal that VraSR is a critical regulatory system that contributes to the survival of *S. suis* and its ability to defend against host innate immunity.

## Introduction

*Streptococcus suis* is a major porcine pathogen that is associated with serious diseases including septicemia, arthritis, endocarditis, pneumonia, and meningitis, as well as sudden death, and it leads to severe economic losses worldwide [1]. As a zoonotic pathogen, *S. suis* can be communicated to humans through direct contact with infected pigs or contaminated pig byproducts, resulting in meningitis and streptococcal toxic shock-like syndrome (STSLS) [2]. So far, cases of human *S. suis* infection have been reported predominantly in several Asian countries, followed by most of Western Europe, Canada, the United States, Argentina, Chile, Australia and New Zealand, with Asian countries accounting for more than 90% of all reported cases from 1 January 2002 to 31 December 2013 [1,3]. Among the 33 serotypes, *S. suis* serotype 2 (*S. suis* 2) seems to be the most prevalent and virulent type that is associated with infections in both pigs and humans worldwide [4]. In China, two large-scale outbreaks of severe human infections of *S. suis* 2, reported in 1998 and 2005, raised international concern and highlighted the threat to public health posed by *S. suis* [5]. However, the mechanisms that contribute to the virulence of *S. suis* are as yet poorly understood.

Human polymorphonuclear leukocytes (PMNs; neutrophils) account for a large proportion of all leukocytes in the bloodstream and are indispensable as the primary cellular defense against invading pathogenic microbes. Following infection, PMNs are recruited to the site of infection, phagocytize invasive bacteria, and simultaneously and progressively release reactive oxygen species (ROS) as well as cytotoxic molecules from granules to destroy the ingested microorganisms [6].

To monitor and respond to environmental stimuli, microorganisms use two-component signal transduction systems (TCSs), which are typically composed of a membrane-bound sensor histidine kinase (HK) and a cytoplasmic response regulator (RR), to coordinate a transcriptional response [7]. Furthermore, TCSs have been implicated in virulence factor expression in various bacterial species in response to external stimuli [8–11]. The *S. suis* 2 genome contains 15 putative TCSs along with orphan regulatory systems [12]. To date, RevS, SalK/SalR, CiaRH, VirR/VirS, Ihk/Irr, NisK/NisR, and 1910HK/RR have been identified to be positive regulations of virulence in *S. suis*, while CovR was reported as a negative feedback virulence regulator [13–20]. However, the TCS regulatory mechanisms used by *S. suis* 2 to defend against destruction by the innate immune system have not been directly investigated.

In this study, we used qRT-PCR to determine the transcriptional expression levels of 15 putative TCSs in *S. suis* 2 following stimulations by PMNs. Several TCS RRs were found to be significantly up-regulated, including the uncharacterized TCS SSUSC84_0372hk/0373rr, which is orthologous to the VraSR system standing for vancomycin-resistance-associated sensor and RR of *Staphylococcus aureus* [21]. The VraSR system was shown to regulate genes associated with cell wall biosynthesis in response to cell wall inhibitors and/or cell envelope damage in *S. aureus* [22]. Although VraSR was significantly up-regulated in response to PMN phagocytosis based on a comprehensive DNA microarray analysis of *S. aureus* gene expression [23], the regulation of VraSR in the virulence and innate immune evasion of *S. aureus* and *S. suis* remains unclear.

In this work, we demonstrated that VraSR is an essential TCS that significantly facilitates the resistance of *S. suis* 2 to killing by human blood and PMNs *in vitro* as well as to bacterial clearance within blood and various tissues during systemic infection in mice. Furthermore, VraSR was also found to have no influence on the production of many inflammatory cytokines at an early stage of *S. suis* infection, but the levels of these cytokines sharply decrease later in infection.

## Results

### PMN stimulation induces differential TCS regulator gene expression in *S. suis 2*

**Figure 1.**
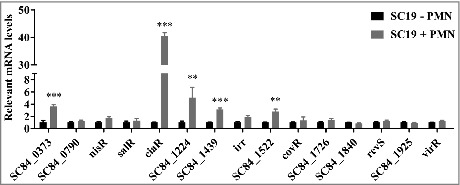
Differential *S. suis* 2 two-component signal transduction system response regulator genes expression within the PMN environment. PMNs were incubated with SC19 for 180 min, after which bacterial RNA was isolated and purified, and expression levels were measured by qRT-PCR. The relative expression level of each gene was normalized to that of the housekeeping genes *proS* and *16S rRNA*. **p < 0.01 and ***p < 0.001 compared to untreated SC19.

To test the idea that TCSs are involved in evading innate host defenses in the pathogen *S. suis*, we determined the expression levels of 15 known RR genes in the virulent *S. suis* strain SC19 after interaction of these cells with human PMNs. Given that there are four orphan regulatory systems that possess only RR in *S. suis* 2, we designed primers to 15 RRs for qRT-PCR to detect the TCS expression levels. We discovered that the transcription level of VraR (SC84_0373), CiaR, SC84_1224, SC84_1439 and SC84_1522 were all significantly increased following exposure to PMNs compared with SC19 incubation alone; in contrast, the other RRs showed little or no response to PMN stimulation (Fig. 1). The CiaRH RR gene expression levels were extremely high, showing a 40.480-fold difference from unstimulated cells, while VraR showed a 3.634-fold increase, SC84_1224 showed a 5.085-fold increase in expression. These data indicate that *S. suis* employs a series of TCSs to respond to PMN-mediated innate immunity.

### Analysis of the *vraSR* gene in *S. suis*

We confirmed the presence of TCS VraSR among *S. suis* strains using NCBI BLAST. The HK *vraS* and RR *vraR* sequences of strain SC84 showed the highest nucleotide sequence identity with the *vraSR* of the other 25 *S. suis* strains (98% to 100%), regardless of whether the strains belonged to different serotypes or genotypes (Table S1). This result demonstrates that the VraSR is present and highly conserved among most *S. suis* strains.

### Construction and characterization of the mutant strain Δ*vraSR*

**Figure 2.**
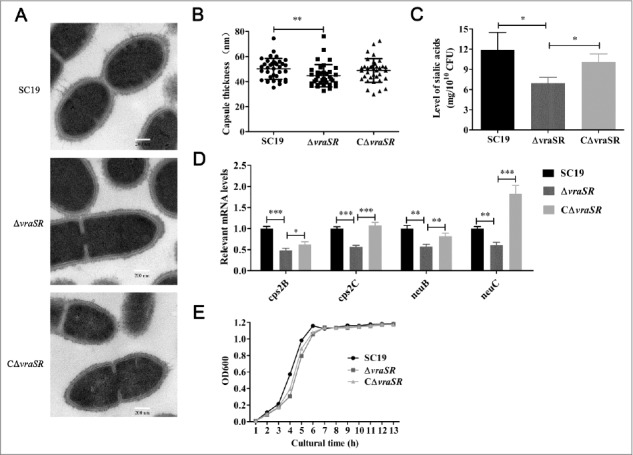
Characterization of mutant strain Δ*vraSR*. (A) Transmission electron micrographs of S. suis. Scale bar: 200 nm. (B) The thickness of the bacterial capsules. Data are the result of 36 bacterial capsules analyzed per sample using Image J software. (C) Concentrations of sialic acid in the S. suis strains. (D) Expression of capsule synthesis-associated genes in vitro. (E) Growth curves for the strains grown in TSB with 10% serum. Bacterial cell density was measured with a spectrophotometer at 600 nm. *p < 0.05, **p < 0.01 and ***p < 0.001 compared to vraSR mutant strain.

We constructed an isogenic *vraSR* gene knockout mutant, Δ*vraSR*, using the strategy of homologous recombination (Fig. S1A). The successful construction of the mutant was confirmed by PCR and RT-PCR of Δ*vraSR* (Fig. S1B and C). Transmission electron microscopy (TEM) measurements revealed that the capsule was thinner in the Δ*vraSR* mutant (44.74 ± 8.9 nm) than in the wild-type (WT) SC19 (50.23 ± 8.4 nm; **p < 0.01) (Fig. 2A and 2B). And the capsule in the complementary strain, CΔ*vraSR*, (48.94 ± 9.5 nm) appeared thicker than the mutant, although this difference was not statistically significant. In contrast compared with SC19 and CΔ*vraSR*, the mutant had significantly lower levels of sialic acid, which is a vital component of *S. suis* capsule, implying that Δ*vraSR* may have a defective capsule (Fig. 2C). The idea that Δ*vraSR* has a thinner capsule is also supported by qRT-PCR results showing that the capsular synthesis-associated genes *cps2B, cps2C, neuB* and *neuC* were significantly down-regulated in Δ*vraSR* compared with WT SC19 and CΔ*vraSR* (Fig. 2D). The growth curves for the WT, Δ*vraSR* and CΔ*vraSR* cultured in TSB medium with 10% serum were compared; although the mutant appeared to have slower growth than the WT strain, this difference was not statistically significant (Fig. 2E).

### Deletion of VraSR attenuates the survival of *S. suis* cells in human blood

**Figure 3.**
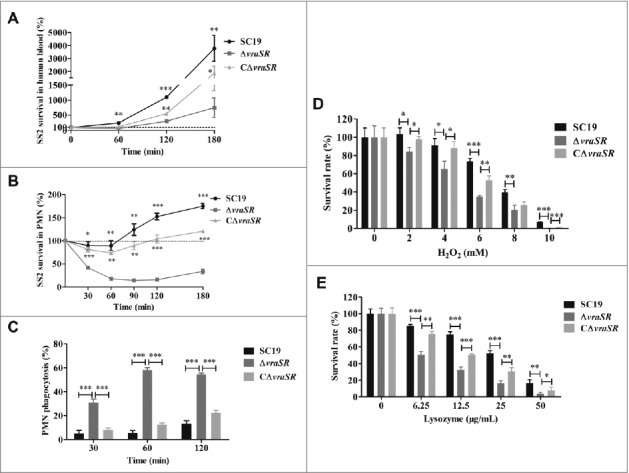
Kinetics of S. suis killing or phagocytosis by human blood, PMNs, and bactericidal substances. (A) Absence of vraSR significantly attenuates the ability of S. suis 2 to survive in human blood. (B) Decreasing survival ability of ΔvraSR over time in the PMN environment. At each time point, PMNs were lysed and viable bacteria were counted on TSA. Results of representative experiments are shown. (C) Phagocytosis of S. suis strains by human PMNs. SC19, ΔvraSR and CΔvraSR were labeled with FITC and incubated with PMNs for different lengths of time. Flow cytometry was used to measure the phagocytosis efficiency. (D) Killing of SC19, ΔvraSR and CΔvraSR S. suis strains by H_2_O_2_. (E) Killing of SC19, ΔvraSR and CΔvraSR S. suis strains by lysozyme. S. suis cultures in exponential phase were incubated with gradient concentrations of H_2_O_2_ or lysozyme at 37 °C for 30 min, and the numbers of viable cells were determined. *p < 0.05, **p < 0.01 and ***p < 0.001 compared to vraSR mutant strain.

After finding that VraR is significantly up-regulated in *S. suis* following stimulation by PMNs, we proposed that VraSR contributes to bacterial survival in blood. To test this hypothesis, we performed a bactericidal assay to examine the ability of the WT SC19 and mutant Δ*vraSR* to survive in healthy whole human blood. Compared with WT SC19, which generally maintained a high growth rate in human blood, the viability of Δ*vraSR* was significantly reduced in human blood after 1 h of incubation (average survival rate: 246.42 ± 58.51% in WT SC19, 123.49% ± 30.47% in the complementary strain CΔ*vraSR* and 69.89% ± 20.30% in the Δ*vraSR* mutant, **p < 0.01). Following this, Δ*vraSR* mutant bacteria grew slowly in human blood (Fig. 3A). This finding supports the idea that the presence of VraSR improves the survival of *S. suis* in human blood.

### VraSR facilitates ability of *S. suis* to resistant to PMN-mediated killing

To determine whether the decreased survival ability of Δ*vraSR* in human blood was due to an increased susceptibility to PMN killing, the role of VraSR in *S. suis* survival in the presence of PMNs was investigated. The resulting time-killing kinetic curve for PMNs, shown in Fig. 3B, illustrates that more than half of the Δ*vraSR* cells were killed by PMNs after 30 min of coincubation, whereas coincubation with PMNs had little effect on the survival of WT SC19 (*p < 0.05 at 30 min and **p < 0.01 at 60 min versus SC19). Furthermore, PMNs could continuously impair the survival ability of the Δ*vraSR* mutant within a certain time range *in vitro*. In comparison, WT SC19 exhibited a strong resistance to PMN-mediated killing and underwent rapid proliferation over time and the complementary strain CΔ*vraSR* also showed a restored ability to defend against PMN-mediated killing. These experiments suggest that VraSR contributes to neutrophil resistance and that the decreased survival of Δ*vraSR* in human blood was due to PMN killing.

### VraSR contributes to bacterial defense against phagocytosis by PMNs

PMN phagocytosis is the primary mechanism by which the host defends against and eliminates invading pathogens. To assess whether the decreased survival of Δ*vraSR* in PMNs was due to increased ingestion by PMNs, the PMN phagocytosis of *S. suis* was analyzed by flow cytometry (Fig. 3C). We found an increased uptake of Δ*vraSR* by PMNs from 30 to 60 min of incubation, followed by no increase in phagocytosis from 60 to 120 min (58.23% ± 1.92% at 60 min vs. 54.40% ± 1.22% at 120 min). However, almost no phagocytosis of WT SC19 was observed within the 60-min incubation. By 120 min of incubation, PMNs had ingested only 13.1% ± 2.61% of SC19 cells. These data indicate that VraSR contributes to the ability of *S. suis* to resist phagocytosis by PMNs.

### Absence of *vraSR* diminishes the resistance of *S. suis* to killing by oxidant and lysozyme

Host cells exercise various strategies to kill invasive bacteria, including the release of ROS during the respiratory burst and cell wall-destabilizing enzymes. We examined the susceptibility of the WT SC19, Δ*vraSR* and CΔ*vraSR* to H_2_O_2_ which is one of the main bactericidal oxidants generated by host phagocytes *in vitro*. The survival of mutant strain Δ*vraSR* was significantly lower compared with the WT upon exposure to H_2_O_2_ at concentrations ranging from 2 mM to 10 mM (Fig. 3D). After 30 min of incubation with 10 mM H_2_O_2_, nearly all the mutant cells were killed, whereas 7.02% of the WT cells survived. Furthermore, we observed a significant decrease in the ability of Δ*vraSR* to survive on exposure to lysozyme, which is an antibacterial protein, and this decreased ability exhibited a concentration-dependent pattern (Fig. 3E). The complementary strain CΔ*vraSR* had a restored WT phenotype of being resistant to H_2_O_2_ and lysozyme killing. These results demonstrate that VraSR in *S. suis* contributes to resistance to multiple antimicrobial substances.

### Contribution of VraSR to cell adhesion

**Figure 4.**
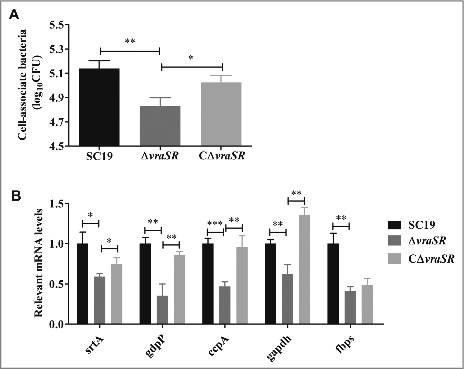
HBMEC cell adhesion assays. (A) Ability of *S. suis* strains to adhere to HBMEC cells. (B) Adhesion-associated gene expression of different strains *in vitro*. Total RNA was extracted from SC19, Δ*vraSR* and CΔ*vraSR* grown in TSB medium with 10% serum at mid-log phase and used for qRT-PCR. Results are shown as the relative expression ratio compared with their expression in the parental strain SC19. These data represents the means of triplicate values. *p < 0.05, **p < 0.01 and ***p < 0.001 compared to *vraSR* mutant strain.

The ability of bacteria to adhere to host tissues is particularly important in disease pathogenesis. In particular, *S. suis* can breach the blood-brain barrier (BBB) to induce host meningitis through the first step to adhere to brain microvascular endothelial cells (BMEC), which constitutes one of the important components of BBB protecting the brain from bacteria in the bloodstream [24,25]. We observed that *S. suis* strains adhere to human BMEC cell line (HBMEC), but significantly fewer Δ*vraSR* were cell-associated compared with the WT SC19 (Fig. 4A). Consistent with this finding, the adhesion-associated genes encoding sortase A (*srtA*), c-di-AMP phosphodiesterase (*gdpP*), catabolite control protein A (*ccpA*), fibronectin- and fibrinogen-binding protein (*fbps*) and glyceraldehyde-3-phosphate dehydrogenase (*gapdh*) were obviously down-regulated in the Δ*vraSR* mutant compared with the WT SC19 (Fig. 4B). Collectively, these data reveal that VraSR promotes the ability of *S. suis* to adhere to HBMEC.

### Δ*vraSR* had attenuated virulence and colonization in a mouse model

**Figure 5.**
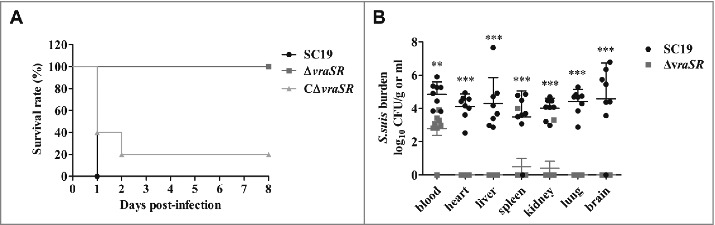
Virulence of Δ*vraSR* in a BALB/c mouse model. (A) Survival curves for mice infected with *S. suis* strains. Differences in the survival rates between SC19 and the mutant strains were analyzed with the log-rank test (**p < 0.01). (B) Bacterial burdens in the blood (CFU/mL of blood), and in the heart, liver, spleen, lung, kidney and brain (CFU/g of tissue). **p < 0.01 and ***p < 0.001 compared to *vraSR* mutant strain.

To determine whether VraSR is involved in *S. suis* virulence *in vivo*, a BALB/c mouse model was used to test the virulence of *S. suis* strains SC19, Δ*vraSR* and CΔ*vraSR* (Fig. 5A). All the mice challenged intraperitoneally with the WT strain suffered morbidity and died within 18 h post-infection (PI), and most of the mice (4/5) infected with CΔ*vraSR* died within 48 h after infection; in contrast, all of the Δ*vraSR*-infected mice survived.

To further evaluate the virulence attenuation of mutant strain Δ*vraSR*, bacterial burden experiments were performed. We harvested the blood and organic tissues of the infected mice and enumerated the bacterial burden at 72 h PI (Fig. 5B). Significant differences in the number of recovered bacteria from blood were found between SC19 and Δ*vraSR* (**p < 0.01). Furthermore, greater numbers of bacteria were recovered from the heart, liver, spleen, lung, kidney and brain of mice infected with WT SC19 compared with the Δ*vraSR* mutant, for which bacteria were generally barely detectable. Taken together, these results reveal that VraSR contributes to *S. suis* virulence *in vivo*.

### VraSR has no influence on inflammatory cytokine production at an early stage of infection

**Figure 6.**
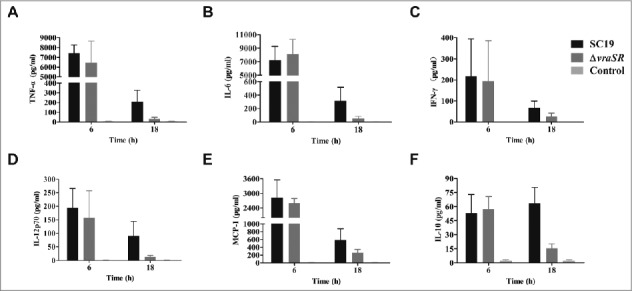
Production of inflammatory cytokines *in vivo* during pathogenic *S. suis* 2 infection. BALB/c mice were injected intraperitoneally with 5 × 10^7^ CFU of SC19 or Δ*vraSR* for 6 h or 18 h. Serum was harvested at each time point post-infection and the concentrations of cytokines were measured by a CBA Mouse Inflammation Kit. (A–F) Concentrations of TNF-α (A), IL-6 (B), IFN-γ (C), IL12p70 (D), MCP-1 (E), and IL-10 (F) in the serum. Results are displayed from three infected mice per group at each indicated time.

To explore the role of inflammatory cytokines in VraSR-mediated *S. suis* 2 infection, we inoculated mice with a non-lethal dose of WT or Δ*vraSR* and harvested serum at 6 h and 18 h PI. The production levels of pro- and anti-inflammatory cytokines and chemokines were determined with a CBA Mouse Inflammation Kit (Fig. 6). Surprisingly, TNF-α, IL-6, IFN-γ, IL-12p70, and MCP-1 exhibited significantly higher production in mice infected with either SC19 or the mutant Δ*vraSR* compared with uninfected controls at 6 h PI, and there was almost no difference in the average concentration of these cytokines between the two groups. At 18 h PI, the two infected groups both had reduced levels of the cytokines described above compared with the corresponding levels at 6 h PI. However, the serum levels of cytokines in Δ*vraSR*-infected mice decreased more sharply than those in SC19-infected mice. We also measured the production of the anti-inflammatory cytokine IL-10. In SC19-infected mice, the serum levels of IL-10 gradually increased from 6 to 18 h PI, whereas in Δ*vraSR*-infected mice, IL-10 levels rapidly decreased from levels similar to those of SC19-infected mice at 6 h PI to nearly basal levels at 18 h PI (**p < 0.01). Thus the VraSR-mediated survival of mice within 18 h may be explained by diminished inflammatory cytokine production.

### Discussion

As an important swine pathogen responsible for heavy economic losses and public health problems around the world, *S. suis* 2 is characterized by its abilities to defend against the host innate immune response, proliferate in human blood and disseminate to vital organs [26]. One mechanism that microorganisms use to sense and respond to external stimuli is the use of TCSs, some of which (e.g., Ihk/Irr in *Streptococcus pyogenes* and SaeR/S in *S. aureus*) have been identified to be essential for innate immune evasion [23,27]. As such, it is important to investigate the direct and indirect response regulations of TCSs in *S. suis* 2 to that act to prolong survival in human neutrophils.

This study provides evidence that *S. suis* 2 use multiple TCS regulatory systems to promote its survival during host infection. The CiaRH in *Streptococcus pneumoniae* has evolved to maintain high levels of gene expression under various conditions rather than responding strongly to a particular signal, and our results are consistent with this observation [28]. Moreover, findings in mouse and piglet models suggest that CiaRH also contributes to the virulence of *S. suis* 2 [16]. The SC84_1223hk/1224rr is a homologue to the YycFG of *S. aureus*, which is essential for the viability of certain Gram-positive bacterial pathogens including *S. suis* 2 [29–32]. As the third most-up-regulated TCS RR gene in PMN-stimulated *S. suis* 2, VraSR positively regulates cell wall biosynthesis, and it can be induced by exposure to cell wall-inhibitory antibiotics and antimicrobial peptides in *S. aureus* [21]. Although it is well established that VraSR can sense cell-envelope stresses, little is known about its function in *S. aureus*, or indeed in *S. suis* 2. Compared with the other significant up-regulated RRs, which may also play an important role in the pathogenesis of *S. suis*, VraSR may have a more direct regulation of *S. suis* 2 to defend against destruction by the host immune system.

As expected, we found that VraSR contributes to the survival of *S. suis* in human blood. Since neutrophils are the predominant phagocytes in human blood, we then conducted studies on the interaction between PMNs and *S. suis* 2, the results of which demonstrated that the absence of VraSR in SC19 significantly reduced the ability of *S. suis* to survive within PMNs and made the bacteria more susceptible to ingestion by PMNs *in vitro*. These findings clearly indicate that *S. suis* 2 uses VraSR to initiate a regulatory response to counteract neutrophil defenses, hence increasing the chances of survival in the host. PMNs circulate in the blood for a few hours and effectively kill bacteria at sites of infection, particularly within tissues [33,34]. In agreement with these, a significantly higher bacterial load was found in various organs of SC19-infected mice at 3 days PI, compared with the barely detected bacterial load in Δ*vraSR*-infected mice. The attenuated virulence of Δ*vraSR* also suggests that VraSR plays a crucial role in the pathogenicity of *S. suis* 2. However, the exact molecular mechanism by which VraSR increases bacterial survival and defends against phagocytosis remains elusive. We noted that VraSR complementation reversed some of the effects of the *vraSR* deletion but did not completely restore the phenotype of the WT strain. Given that the restoration of *vraSR* expression was driven by an inserted plasmid and by an inducible promoter instead of its native promoter, the gene expression and regulation of *vraSR* may not be precisely the same as that of the WT strain under several conditions. Nevertheless, the *vraSR* expression level in CΔ*vraSR* was higher than in Δ*vraSR*.

Effectively avoiding neutrophil phagocytosis and post-phagocytosis killing is likely a critical survival strategy used by pathogenic *S. suis* 2. PMNs recognize and ingest bacterial pathogens typically aided by complement and antibody receptors present on the cell surface [35]. It is well known that the capsular polysaccharide can protect bacteria from phagocytic cell-mediated phagocytosis and prevent opsonization by complement in many different bacterial species [6,36–38]. The increased PMN phagocytosis of Δ*vraSR* compared with SC19 may be due to the thinner capsule, lower sialic acid level, and lower expression of capsule synthesis-related genes in the mutant strain. Consistent with our results, *Streptococcus iniae* have been reported to use *sivS/R* to regulate capsule expression affecting its ability to evade phagocytosis [39]. Following phagocytosis, ingested bacteria are exposed to a high concentration of bactericidal substances such as ROS, antimicrobial peptides and proteases in the phagocytic vacuole [35]. In this study, we detected a statistically significant, concentration-dependent, increase in H_2_O_2_- and lysozyme-killing of the Δ*vraSR* mutant compared with the WT. This finding is consistent with the result of a previous study in which the VraSR was found to participate in regulation of the *msr*A1 gene in PMN-ingested *S. aureus* [40]. Methionine sulfoxide reductase (Msr) is an oxidant repair system that has been demonstrated to contribute to the staphylococcal response to oxidative attack and PMNs [40]. It is not surprising that the absence of VraSR increases the sensitivity to lysozyme, which functions by disrupting cell walls to kill phagocytized bacteria [22]. Similarly, *S. pyogenes* manipulates Ihk/Irr to influence genes involved in the detoxification of ROS and in cell wall synthesis to resist killing by neutrophils [27], suggesting that the ability to endure ROS and bactericidal components contributes to pathogen survival.

Inflammatory cytokine production is an important factor determining whether the immune system is able to provide effective protection against invasive pathogens [41]. *S. suis* 2 has the ability to stimulate abundant pro-inflammatory cytokine production leading to STSLS and high levels of TNF-α, IL-6, IL-12p70, IFN-γ and CCL2 (MCP-1) *in vivo* might be responsible in part for the sudden death of animals within 24 h PI [42]. Neutrophil activation can be induced by cytokines and chemokines such as TNF-α, IL-6 and IFN-γ [43]. As there was no difference in inflammatory cytokine levels at 6 h PI, particularly for IFN-γ, which is known to stimulate many effector functions of mononuclear phagocytes [44], it is likely that the number of activated neutrophils induced by cytokines is similar between SC19- and Δ*vraSR*-infected mice at an early stage of infection. Furthermore, the levels of MCP-1 and IL-6 produced by PMNs stimulated by WT and Δ*vraSR* for 18 h *in vitro* were not significantly different (Fig. S2). MCP-1 is a chemotactic protein that recruits more PMNs and modulates neutrophil functions. The neutrophil activation process occurs on many neutrophil function including phagocytosis, cytokine secretion, degranulation, and oxidative and non-oxidative microbicidal activities [43]. Taken together, the increased sensitivity of Δ*vraSR* to PMNs in our study may explain why cytokine production (including anti-inflammatory IL-10) in Δ*vraSR*-infected mice was significantly decreased at 18 h PI to nearly the baseline levels observed in the non-infected controls. In contrast, IL-10 levels were increased in the serum of mice infected with SC19, reflecting a severe inflammatory response that was likely responsible for the death within 18 h PI of the mice infected with SC19 [42]. Collectively, the lack of regulation of cytokine levels by VraSR at the early stage of infection contributes to bacterial clearance.

Interestingly, the Δ*vraSR* mutant showed a reduction in adherence to HBMECs, accompanied by a thinner capsule, compared with WT SC19, which is contradictory to the findings of previous studies showing that non-encapsulated bacteria exhibit higher adherence to cells [45]. Given that the capsule was thinner rather than absent in the mutant, we hypothesize that the absence of VraSR may have a more significant effect on adherence than the thinner capsule. These findings are in accordance with the observed down-regulation of a series of reported adhesion-associated genes, such as *ccpA*, *fbps*, *gapdh*, *gdpP* and *srtA* (Fig. 4B), which also contribute to the virulence in *S. suis* [46–50].

In summary, we detected multiple TCSs involved in bacterial pathogenesis. Using *in vitro* and *in vivo* models of systemic infection, our study highlighted a novel TCS, VraSR, which contributes to the virulence of *S. suis* and is vital for *S. suis* evasion of host innate immune defenses. This study provides a foundation for further research on the precise molecular mechanisms underlying VraSR-regulated immune evasion by *S. suis* 2.

## Materials and methods

### Human blood and serum

The healthy donors who provided blood and serum for this study at the Huazhong Agricultural University Hospital provided written informed consent prior to participation. All studies were conducted in accordance with approval obtained from the Medical Ethics Committee of the Huazhong Agricultural University Hospital.

### Bacterial strains, plasmids, cell line, and growth conditions

The bacterial strains and plasmids used in this study are shown in Table S2. The virulent *S. suis* strain SC19 was isolated from a diseased piglet during the Chinese *S. suis* 2 outbreak in 2005 [51]. All *S. suis* strains were grown in Tryptic Soy Broth (TSB; BD) or plated on Tryptic Soy Agar (TSA; BD) with 10% (v/v) fetal bovine serum (FBS) at 37 °C. Spectinomycin (100 µg/mL) was incorporated into the growth medium when required. *Escherichia coli* strains were cultured in LB broth or plated on LB agar with a concentration of 50 µg/mL spectinomycin.

The HBMEC cell line was kindly provided by Prof. Kwang Sik Kim, Johns Hopkins University School of Medicine, and shared by Dr. Xiangru Wang, Huazhong Agricultural University [52,53]. HBMECs were cultured in RPMI 1640 medium with 10% FBS, 2 mM L-glutamine, 1 mM sodium pyruvate, essential amino acids, nonessential amino acids, vitamins, and penicillin and streptomycin (100 U/mL) at 37 °C with 5% CO_2_.

### Construction of the mutant and complementary strain of *S. suis 2*

To test the regulation of VraSR in the pathogenesis of *S. suis* 2, the HK gene *vraS* and RR gene *vraR* of VraSR simultaneous deletion strain was constructed through homologous recombination using a thermosensitive suicide vector pSET4s [54]. The primers used in this study are listed in Table S3. PCR was used to amplify the upstream and downstream regions of *vraSR*. Corresponding restriction enzyme digested PCR fragments were cloned into pSET4s to construct the *vraSR* deletion vector pSET4s::*vraSR*. To obtain the isogenic mutant Δ*vraSR*, the recombinant plasmid was electroporated into parental strain SC19 competent cells. The mutant strain was selected on TSA according to its sensitivity to spectinomycin and was confirmed by PCR and DNA sequencing. A complementary strain of *vraSR* was constructed as described previously [55]. The coding sequence of *vraSR* was cloned into pSET2 to generate the recombinant plasmid pSET2::*vraSR*. The complementary strain was acquired by transforming pSET2::*vraSR* into mutant strain Δ*vraSR*.

### RNA extraction and qRT-PCR assays

Human neutrophils were isolated from the sodium citrate venous blood of healthy donors using human PMN isolation solution (TBD Science) according to the manufacturer's specifications. TCS RR gene expression levels in *S. suis* within the PMN environment were analyzed as previously described with several modifications [56]. PMNs (10^6^ cells) were combined with 10^8^ CFU of opsonized *S. suis* 2 in 12-well plates and incubated at 37°C with 5% CO_2_ for 180 min. At the indicated time, samples were treated with 1% saponin for 20 min on ice to release phagocytosed bacteria. Total RNA of *S. suis* 2 was isolated using a Bacterial Total RNA Isolation Kit (Sangon Biotech), and contaminating host RNA was removed with a MICROB*Enrich*™ Purification of Bacterial RNA Kit (Ambion). For qRT-PCR, HiScript® II qRT SuperMix (Vazyme) was used to remove any residual DNA from the RNA and synthesize cDNA, and AceQ qPCR SYBR Green Master Mix (Vazyme) was used to amplify the cDNA by PCR in which two housekeeping genes (*proS* and *16S rRNA*) were used as the internal references [18,57]. The ABI ViiA 7 RT-PCR System was used for qRT-PCR, and the corresponding data were analyzed with ExpressionSuite Software (Life Technologies).

### Transmission electron microscopy (TEM)

TEM was utilized to study the microstructures of *S. suis* following *vraSR* deletion [58]. WT SC19, mutant Δ*vraSR* and complementary strain CΔ*vraSR* cells harvested at mid-log growth phase were fixed with 2.5% glutaraldehyde overnight at 4°C, postfixed with 1% osmium tetroxide for 2h, dehydrated in ethanol and embedded in epoxy resin, after which the cell morphologies were examined on a model Tecnai G^2^ 20 TWIN TEM (FEI Company) at 200 kV. High-magnification images were captured with a digital camera system.

### Bacterial sialic acid extraction and concentration measurement

For the preparation of sialic acid, the thiobarbituric acid method of Warren-Aminoff was used with several modifications [59,60]. The bacteria were grown in TSB with 10% (v/v) FBS for 5 h at 37 °C and pelleted by centrifugation, resuspended in phosphate-buffered saline (PBS), and adjusted to 1 × 10^10^ CFU/mL. Bacteria in suspension (1mL) were lyophilized and then incubated with 500 µL of hot acid (0.4 M H_2_SO_4_, 80 °C) for 1 h to release sialic acid through the hydrolysis performed on lyophilized bacteria. The concentrations of sialic acid were measured by a sialic acid assay kit (Nanjing Jiancheng Bioengineering Institute) according to the specifications of the manufacturer.

### Oxidative stress and lysozyme killing assays

To evaluate the sensitivity of *S. suis* to oxidative stress and lysozyme, bacteria were incubated with serial dilutions of H_2_O_2_ or chicken egg white lysozyme (Sigma), and survival assays were performed as previously reported [18]. Bacteria were harvested at exponential phase and suspended in TSB with 10% serum at 10^6^ CFU/mL. The sensitivity was examined by exposure of the bacteria to varied concentrations of H_2_O_2_ (0 to 10 mM) or lysozyme (0 to 50 µg/mL) at 37 °C for 30 min. The survival rate was calculated based on the number of viable bacteria on the TSA plate before and after exposure to those microbicidal substances.

### Cells adhesion assay

HBMEC adhesion experiments were performed as previously described [18]. Briefly, exponential phase bacteria were collected and resuspended in RPMI 1640 medium to a concentration of 10^6^ CFU/mL. Monolayer cells (10^5^ cells per well) grown in 24-well culture plates were incubated with bacteria at a MOI of 10:1 for 3 h at 37 °C with 5% CO_2_. The cells were then washed with PBS and lysed with saponin. Final counts of cell-associated bacteria were based on the number of viable bacteria on the TSA plate before and after incubation with HBMECs.

### Human blood killing assay

To investigate the role of Δ*vraSR* in human blood, a blood bactericidal experiment was performed as previously published [61]. Briefly, log-phase bacteria were collected and resuspended in PBS after being washed three times with PBS, and then 100 µL (1 × 10^4^ CFU/mL) of bacteria were mixed into 900 µL of fresh heparinized human venous blood from healthy donors. The mixtures were incubated at 37 °C with moderate rotation. At the indicated times, 100 µL of the cultured mixtures were diluted and plated on TSA. The percentage of bacterial survival in blood was calculated by comparing the number of surviving bacteria at each time point to the original number of bacteria.

### Human PMN bactericidal assay

Human PMN bactericidal experiments were performed as described previously with several modifications [62]. Briefly, exponential phase bacteria were diluted to a concentration of 10^5^ CFU/mL in nonimmune human serum and opsonized for 30 min at 37 °C. PMNs (100 µL, 10^6^ cells/mL) were combined with opsonized bacteria (100 µL, 10^5^ CFU/mL) in 96-well plates (MOI = 1:10) and centrifuged at 400 × g for 5 min at 4 °C. The cells then were incubated at 37 °C with 5% CO_2_. Samples were taken out at 30, 60, 90, 120, and 180 min and immediately lysed with saponin. The percentage of *S. suis* 2 survival in PMNs was measured using the equation: (CFU_PMN+_/CFU_PMN-_) × 100%.

### Human PMN phagocytosis experiments

Phagocytosis of *S. suis* by human PMNs was performed as described previously with minor modifications [56]. Bacteria were suspended in PBS at 10^9^ CFU/mL, labeled with 5 µg/mL of FITC in 0.1 M carbonate-bicarbonate buffer solution (pH 9.4) at 37 °C for 30 min, and washed with PBS to remove any residual FITC. Labeled bacteria were opsonized in nonimmune human serum for 30 min at 37 °C, diluted to 10^7^ CFU/mL in RPMI 1640, and chilled on ice until use. PMNs (100 µL, 10^6^ cells/mL) were mixed with *S. suis* (100 µL, 10^7^ CFU/mL) in 96-well plates (MOI = 10:1). The plates were centrifuged at 400 × g for 5 min at 4 °C and then incubated at 37 °C with 5% CO_2_. At the indicated times, samples were immediately analyzed by flow cytometry, and the PMN phagocytosis efficiency was determined based on the percentage of FITC-positive PMNs containing fluorescent bacteria.

### Experimental mouse infection

All the animals used in this study and all animal experiments were approved by Committee on the Ethics of Animal Experiments of the College of Veterinary Medicine, Huazhong Agricultural University, and all efforts were made to minimize suffering.

To assess the effects of VraSR in *S. suis* virulence, 5-week-old female BALB/c mice were divided into three groups (5 mice per group) and injected intraperitoneally with 2 × 10^8^ CFU of WT SC19, Δ*vraSR*, or complemented strain CΔ*vraSR*. Survival and clinical signs were observed daily for up to 8 days PI.

Bacterial burdens in the blood and various organs were determined as described previously [62]. Mice (8 mice per group) were inoculated via the intraperitoneal route with 5 × 10^7^ CFU of SC19 or the Δ*vraSR* mutant. At 72 h PI, mice were anesthetized and then exsanguinated to determine the *S. suis* load in the blood. The liver, spleen, kidney, heart, lung and brain were then excised to determine the bacterial burden in each organ, and all the samples were homogenized in PBS after weighing. The blood and organ homogenates were diluted and plated on TSA.

### Cytokine measurement assays

Mice were inoculated with 5 × 10^7^ CFU of SC19 or mutant Δ*vraSR* as described above. At each indicated time, mouse blood was collected by cardiac puncture. Serum was separated by centrifugation at 3000 × g for 5 min and stored at −80 °C until analysis.

To investigate the release of cytokines in neutrophils directly stimulated by *S. suis* strains, cytokines analysis of PMNs incubated with *S. suis* were performed as previously described with several modifications [63]. Mouse PMNs were isolated with a Mouse PMN Isolation Solution (TBD Science) follow the instructions. PMNs (100 µL, 10^6^ cells/mL) were mixed with *S. suis* cells (100 µL, 10^5^ CFU/mL) in 96-well plates, and PMNs with added LPS (final concentration 1 µg/mL) were used as the positive control, while PMNs with only RPMI 1640 medium served as the negative control. After 18 h of stimulation, the supernatants were collected, and their cytokine levels were analyzed.

The concentration of inflammatory cytokines in the serum or culture supernatants was measured on a BD FACSVerse flow cytometer using a CBA mouse inflammation kit (BD Biosciences) according to the specifications. The data were analyzed with FCAP Array software.

### Statistical analysis

All statistical analyses were performed with GraphPad Prism 5 software. Differences between the WT strain and mutant strains were analyzed using an unpaired, two-tailed Student's *t*-test with a 95% confidence interval. Mouse survival rates were analyzed with the log-rank test. Unless otherwise specified, data are presented as the mean ± SD. For all tests, *p* < 0.05 was considered statistically significant.

## Supplementary Material

KVIR_A_1428519_supplementary_material.docx
