# Supplementary material for: The VraSR regulatory system contributes to virulence in Streptococcus suis via resistance to innate immune defenses
Source: Virulence. 2018 Apr 24;9(1):771–82. doi: 10.1080/21505594.2018.1428519 (PMC5955479; doi:10.1080/21505594.2018.1428519)
Supplement: KVIR_A_1428519_supplementary_material.docx [file kvir-09-01-1428519-s001.docx]

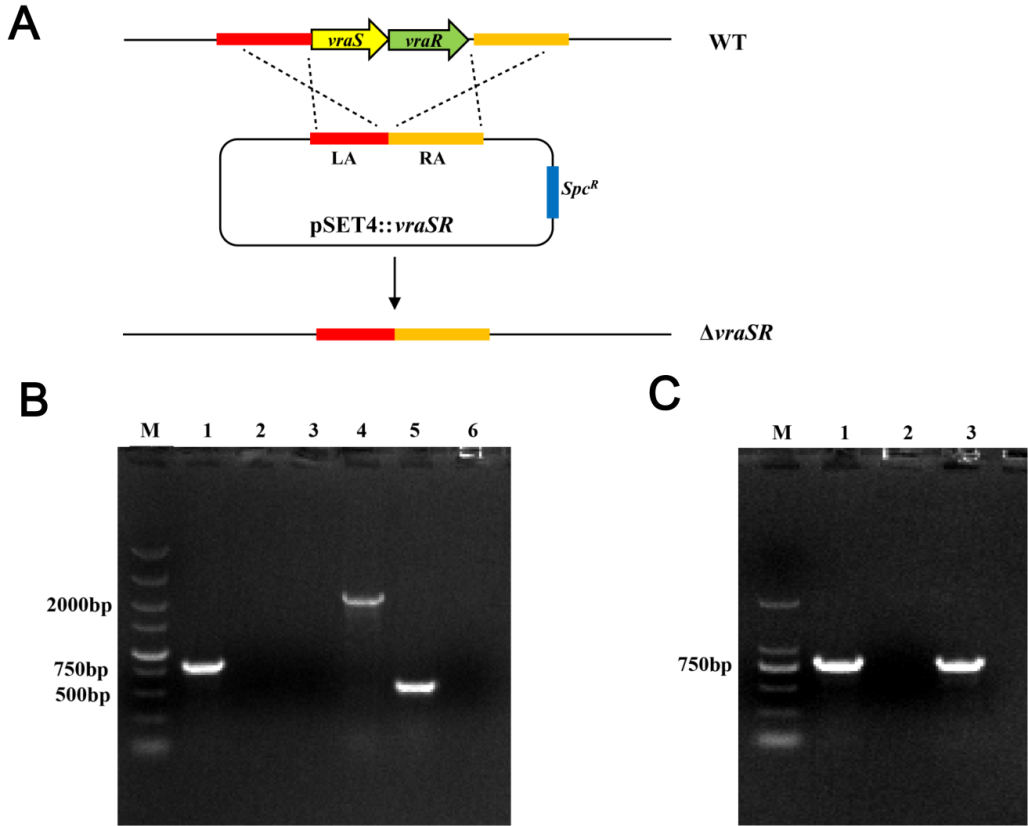


**Supplementary Figure 1. Construction and confirmation analysis of the knockout mutant strain Δ*vraSR*.** (A) Strategy for knockout of *vraS* and *vraR* genes in *S. suis* wild type by homologous recombination. The closed circle indicates the recombinant pSET4s vector. LA and RA are represented the left and the flanking regions of *vraSR*. (B) Confirmation the mutant Δ*vraSR* by PCR. Lanes 1-3 represent the amplification of *vraSR* using the primer pair *vraSR*-F and *vraSR*-R. Lanes 4-6 represent the amplification of upstream and downstream gene including *vraSR* using the primer pair *vraSR*-Up and *vraSR*-Down. Lanes 1and 4 use the genomic DNA of SC19 as templates, whereas Lanes 2 and 5 use genomic DNA of Δ*vraSR* as templates. Lanes 3 and 6 represent the negative control. (C) Confirmation of Δ*vraSR* and CΔ*vraSR* by RT-PCR. Lanes 1-3 represent the amplification of *vraSR* using primer pair *vraSR*-F and *vraSR*-R. Lane 1 uses cDNA SC19 as template, whereas lane 2 uses cDNA of Δ*vraSR* as template and lane 3 uses cDNA of CΔ*vraSR* as template.


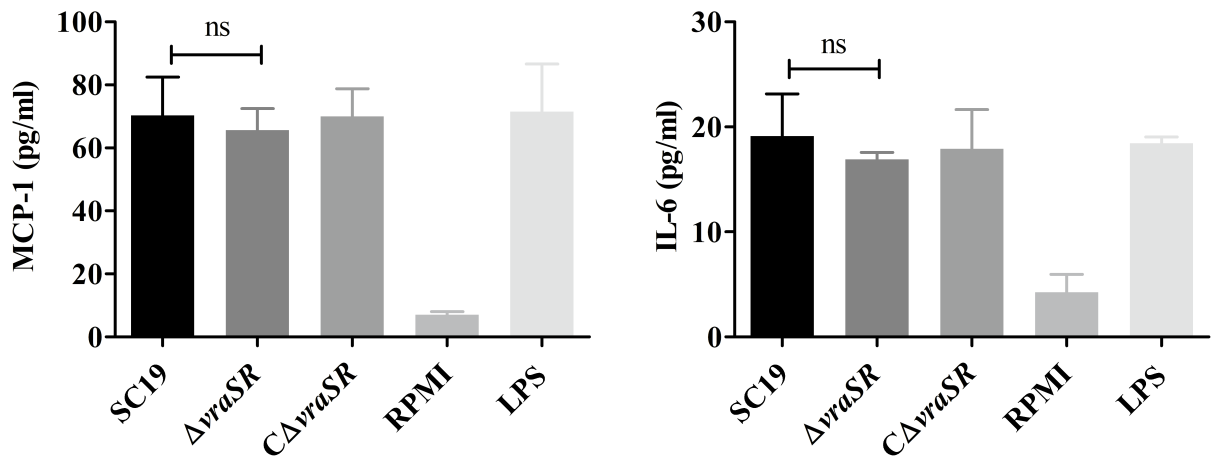


Figure S2. MCP-1 and IL-6 release by mouse neutrophils stimulated by *S. suis* 2. PMNs were incubated with *S. suis* 2 (MOI=1:10) at 37 ℃ with 5% CO_2_ for 18 h and release of cytokines were determined by a CBA Mouse Inflammation Kit. RPMI 1640 medium and lipopolysaccharide (LPS) were performed as negative control and positive control respectively.
